# 99m-Technetium binding site in bone marrow mononuclear cells

**DOI:** 10.1186/s13287-015-0107-0

**Published:** 2015-06-04

**Authors:** Grazielle Dias Suhett, Sergio Augusto Lopes de Souza, Adriana Bastos Carvalho, Rachel de Pinho Rachid, Narcisa Leal da Cunha-E-Silva, Antonio Carlos Campos de Carvalho, Lea Mirian Barbosa da Fonseca, Regina Coeli dos Santos Goldenberg, Bianca Gutfilen

**Affiliations:** Laboratório de Cardiologia Celular e Molecular, Instituto de Biofísica Carlos Chagas Filho, Universidade Federal do Rio de Janeiro (UFRJ), Av. Carlos Chagas Filho, 373, Bloco G. Ilha do Fundão, Cidade Universitária, Rio de Janeiro, 21941-902 Brasil; Departamento de Radiologia, Hospital Universitário Clementino Fraga Filho, Universidade Federal do Rio de Janeiro, Rua Prof. Rodolpho Paulo Rocco, 255. Ilha do Fundão, Cidade Universitária, Rio de Janeiro, 21941-913 Brasil; Laboratório de Ultraestrutura Celular Hertha Meyer, Instituto de Biofísica Carlos Chagas Filho, Universidade Federal do Rio de Janeiro (UFRJ), Av. Carlos Chagas Filho, 373, Bloco G. Ilha do Fundão, Cidade Universitária, Rio de Janeiro, 21941-902 Brasil

## Abstract

**Introduction:**

The increasing interest in 99m-technetium (^99m^Tc)-labeled stem cells encouraged us to study the ^99m^Tc binding sites in stem cell compartments.

**Methods:**

Bone marrow mononuclear cells were collected from femurs and tibia of rats. Cells were labeled with ^99m^Tc by a direct method, in which reduced molecules react with ^99m^Tc with the use of chelating agents, and lysed carefully in an ultrasonic apparatus. The organelles were separated by means of differential centrifugation. At the end of this procedure, supernatants and pellets were counted, and the percentages of radioactivity (in megabecquerels) bound to the different cellular fractions were determined. Percentages were calculated by dividing the radioactivity in each fraction by total radioactivity in the sample. The pellets were separated and characterized by their morphology on electron microscopy.

**Results:**

The labeling procedure did not affect viability of bone marrow mononuclear cells. Radioactivity distributions in bone marrow mononuclear cell organelles, obtained in five independent experiments, were approximately 38.5 % in the nuclei-rich fraction, 5.3 % in the mitochondria-rich fraction, 2.2 % in microsomes, and 54 % in the cytosol. Our results showed that most of the radioactivity remained in the cytosol; therefore, this is an intracellular labeling procedure that has ribosomes unbound to membrane and soluble molecules as targets. However, approximately 39 % of the radioactivity remained bound to the nuclei-rich fraction. To confirm that cell disruption and organelle separation were efficient, transmission electron microscopy assays of all pellets were performed.

**Conclusions:**

Our results showed that most of the radioactivity was present in the cytosol fraction. More studies to elucidate the mechanisms involved in the cellular uptake of ^99m^Tc in bone marrow cells are ongoing.

## Introduction

Stem cells derived from different sources hold therapeutic potential for the treatment of many diseases [1, 2]. Tracking these cells in vivo represents an ongoing challenge in cell-based therapies [3, 4]. Advanced technology, such as non-invasive imaging of transplanted cells to monitor their fate in vivo, has been extensively used and may provide important information for understanding the mechanism of action of these therapies [5, 6].

Cell tracking for in vivo detection of grafted cells can be performed by ultrasound, optical imaging, magnetic resonance imaging, micro-computed tomography imaging, and nuclear medicine techniques [7, 8]. In general, the ideal imaging modality is determined by the specificity, sensitivity, resolution, and radiation exposure of individual modalities [9].

All of the available imaging methods are based on different principles, having different properties and limitations. According to Frangioni and Hajjar [10], there are eight characteristics of an ideal marker for stem cell tracking: to be biocompatible, safe, and non-toxic; not to produce genetic modification in the stem cell; to allow quantification of exact cell number at any anatomic location; to detect a small amount of cells; to be minimally or not diluted by cell division; to be minimally or not transferred to non-stem cells; to be detected by non-invasive imaging technology for months to years; and not demand contrast agent injection. Although some markers have many of these characteristics, none of them fulfills all eight of the criteria presented above.

The fluorescent dyes are the most used to track injected cells in pre-clinical trials. Among these fluorescent dyes, the most commonly used are DAPI (4′6-diamidino-2-phenylndole) and Hoechst 33342 (bis-benzimide). They permeate through the plasma membrane and have strong affinity to DNA, binding predominantly to the nucleus [11, 12]. Another fluorescent dye widely used is Dil, a long chain of carbocynine that, unlike DAPI and Hoechst, binds to plasma membrane [13]. An advantage of labeling cells with these dyes is to find the possible location and integration of the cells to the tissue; however, these markers are diluted with each cell division. Furthermore, fluorescent dyes can be visualized only by microscopy, which is not compatible with in vivo analysis*.*

More recently, bioluminescence imaging has been extensively used to detect the biodistribution of transplanted cells in live small animals. The clinical application is restricted since this technique is reporter gene-based [14].

Nuclear medicine is characterized by an excellent in vivo sensitivity and whole-body imaging capabilities. This technique is suitable for tracking cells in both laboratory [15, 16] and clinical [17–20] settings. Radioisotope cell labeling is a well-established method, and the most commonly used radioisotopes are 111-indium or 99m-technetium (^99m^Tc) or 18F-fluorodeoxyglycose [18F]FDG [21].

The majority of stem cell clinical trials use bone marrow mononuclear cells (BMMCs), and ^99m^Tc is the radionuclide predominantly employed. Our research group has successfully labeled stem cells with ^99m^Tc. We use a simple and efficient labeling technique that maintains cell viability and reaches high labeling efficiency and stability rates [15, 17, 18, 20, 22, 23].

The increasing interest in ^99m^Tc-labeled stem cells encouraged us to study the binding sites of ^99m^Tc to stem cell compartments. This study investigated the presence of ^99m^Tc taken up by different organelles in BMMCs. Cells were labeled with ^99m^Tc by a direct method previously described by our group [15, 24, 25]. The organelles were separated by means of differential centrifugation and characterized by their morphology on electron microscopy.

## Methods

### Animals

All procedures were performed in accordance with the Guide for Care and Use of Laboratory Animals (Department of Health and Human Services Publication #NIH 85–23, revised 1996; Office of Science and Health Reports, Bethesda, MD, USA). This study was approved by the Ethics Committee for Animal Use of the Federal University of Rio de Janeiro under number IBCCF 028/2008.

Wistar rats were obtained from Instituto de Biofísica Carlos Chagas Filho (IBCCF) (Rio de Janeiro, Brazil). Animals were housed at a controlled temperature (23 °C) with daily exposure to a 12:12 light-dark cycle.

### Mononuclear cell isolation from bone marrow

Bone marrow cells obtained from Wistar rats were used for cell labeling. Femurs and tibia were harvested, and all adjacent muscle tissue was thoroughly removed. Bone epiphysis was removed, and bone marrow was flushed by using a syringe filled with Dulbecco’s modified Eagle’s medium (DMEM) (Gibco-Invitrogen, Carlsbad, CA, USA). The cell suspension was carefully placed on top of Ficoll Histopaque 1.083 (Sigma-Aldrich, St. Louis, MO, USA) on 15 ml tubes maintaining a proportion of 1:1 in volume. Tubes were centrifuged at 400×*g* for 30 min at room temperature. Mononuclear cells were collected from the interface formed between Ficoll Histopaque and DMEM. Cells were washed in phosphate-buffered saline (PBS) twice and counted in a hemocytometer, and viability was checked by using trypan blue.

### Labeling the cells with ^99m^Tc

The BMMCs were labeled with ^99m^TcO_4_^−^ on the basis of previously published protocols [15, 24, 25]. All the procedures for cell preparation and labeling were carried out in a laminar flow. Briefly, 500 μl of fresh and sterile SnCl_2_ (stannous chloride) solution was added to the cell suspension in saline solution, and the mixture was incubated at room temperature for 10 min. Then 45 MBq of ^99m^TcO_4_^−^ was added, and the incubation continued for another 10 min. After centrifugation (500×*g* for 5 min), the supernatant was removed, and the cells were washed once more with NaCl 0.9 % solution. The pellet was suspended in NaCl 0.9 % solution, and the viability of the labeled cells was assessed by trypan blue exclusion test. Labeling efficiency (percentage) was calculated by the activity in the pellet divided by the sum of the radioactivity in the pellet plus supernatant.

### Differential centrifugation with lysed cells

After labeling and washing procedures, 1 ml of NaCl 0.9 % solution was added, and the cells were carefully disrupted on ice with 10 cycles of 2 sec, with 1 sec of rest between cycles, in an ultrasonic apparatus (GEX 600 Model; Sigma-Aldrich) by using a standard probe (13 mm radiating diameter), operating at 10 % of total amplitude. Disruption procedure was monitored by phase contrast microscopy, and 0.5 ml of each homogenate sample was separated.

Cell homogenate was added to 10 ml of PBS and centrifuged (Beckman Optima™ Ultracentrifuge, model XL-100 K; Beckman Coulter, Pasadena, CA, USA, rotor 90 Ti) at 2000×*g* for 10 min. The pellet, referred to as pellet I, was reserved, and the supernatant was collected (supernatant I) in a new centrifuge tube. A centrifugation of supernatant I at 12,000×*g* for 20 min generated the second pellet (pellet II) and supernatant (supernatant II). Pellet II was reserved, and supernatant II was collected in a new centrifuge tube. A third centrifugation at 100,000×*g* for 60 min resulted in pellet III and supernatant III [24, 26–28]. At the end of this procedure, supernatants and pellets were counted in a well counter (PerkinElmer, Waltham, MA, USA), and the percentages of radioactivity bound to the different cellular fractions were determined.

### Transmission electron microscopy

The three pellets were fixed with 2.5 % glutaraldehyde in 0.1 M sodium cacodylate buffer (pH 7.2). The supernatants were directly fixed by adding glutaraldehyde to the final concentration of 2.5 %.

All samples were prepared for transmission electron microscopy on the basis of previously published protocols [29]. In short, fixed samples were washed with saline solution, adopting the same centrifugation used to obtain the fractions. Washed fractions were post-fixed in 1 % osmium tetroxide, 0.8 % potassium ferrocyanide, and 5 mM calcium chloride in 0.1 M cacodylate buffer (pH 7.2) for 60 min, dehydrated in an acetone series, and embedded in Epoxy resin. Ultrathin sections were stained with 5 % uranyl acetate and lead citrate and observed in a Zeiss 900 transmission electron microscope (Carl Zeiss AG, Oberkochen, Germany) operating at 80 kV.

## Results

**Fig. 1:**
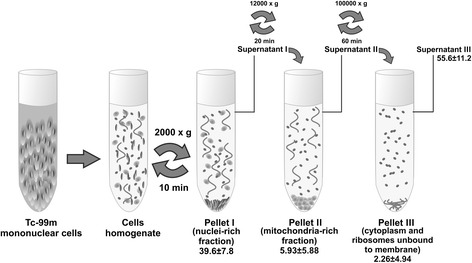
Schematic diagram of differential centrifugation procedure applied to bone marrow mononuclear cells labeled with 99m-technetium (*Tc-99m*). Percentages of radioactivity bound to each fraction are presented as mean ± standard deviation

**Fig. 2:**
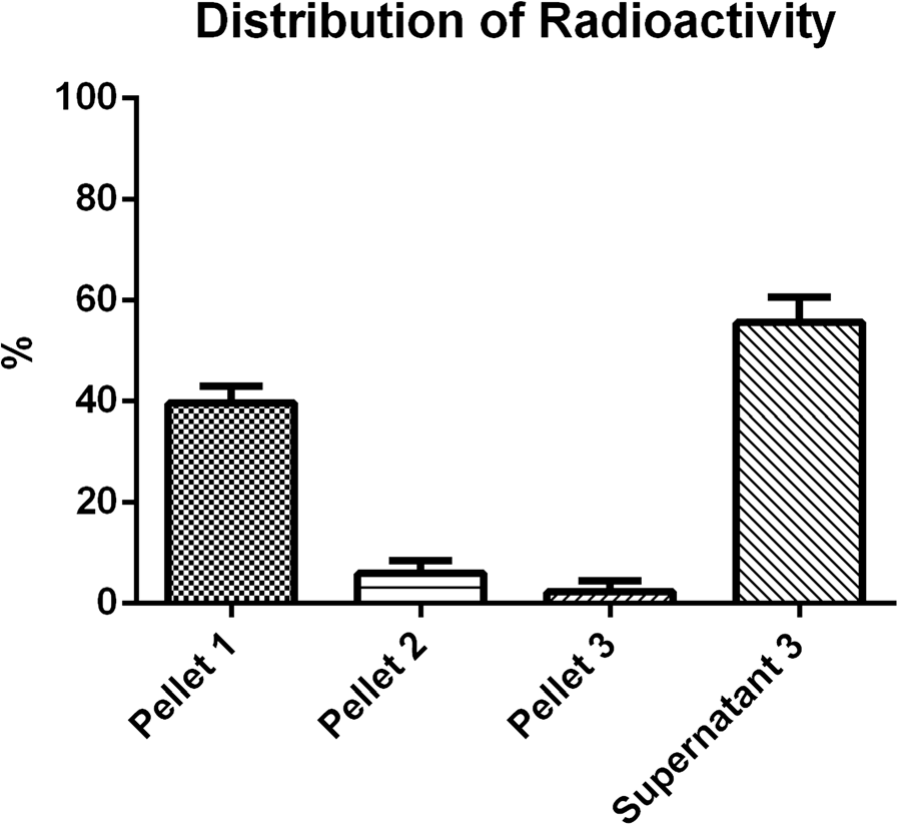
Distribution of 99m-technetium radioactivity in bone marrow mononuclear subcellular fractions. Pellet I: nucleus, cells that were not lysed, and large fragments of plasma membrane; pellet II: mainly mitochondria, liposomes, and peroxisomes; pellet III: microsomal fraction (endoplasmic reticulum and plasma membrane fragments); supernatant III: cytoplasm, ribosomes unbound to membrane, and soluble molecules

The viability of labeled cells was higher than 93 % in all cases. The distribution of ^99m^Tc radioactivity in BMMC organelles is shown in Figs. 1 and 2. The labeling efficiency results are presented as mean ± standard deviation of five independent experiments. Most of the radioactivity remained in the third supernatant (Figs. 1 and 2), which contained the cytosol fraction formed by ribosomes unbound to membrane and soluble molecules. However, approximately 39 % of the radioactivity remained bound to the first pellet, containing the nuclei-rich fraction.

**Fig. 3:**
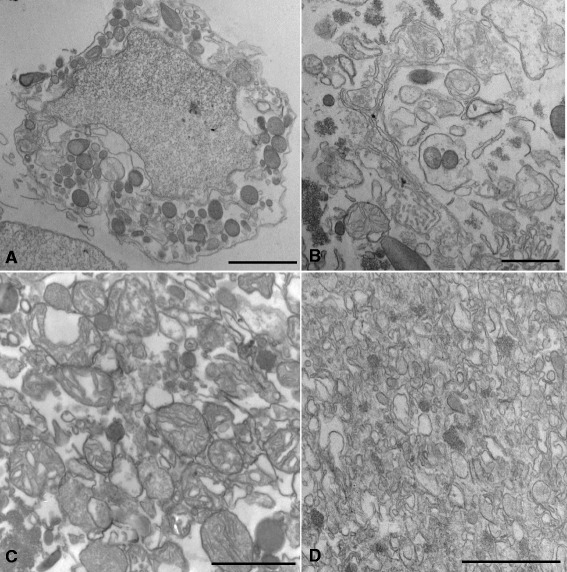
Ultrathin sections of bone marrow mononuclear subcellular fractions observed under transmission electron microscope. Partially disrupted cells (**a**) and large cell fragments (**b**) were found in pellet I, whereas mitochondria and lysosomes were the major component of pellet II (**c**). Many membrane profiles were observed in pellet III (**d**). Bars correspond to 20 μm (a), 10 μm (b), and 1 μm (c and d)

To confirm that cell disruption and organelle separation were efficient, transmission electron microscopy assays of all pellets were performed. Figure 3 shows that the first pellet contained cells that were not lysed (Fig. 3a) and nuclei and large cell fragments (Fig. 3b). The second pellet consisted mainly of mitochondria, lysosomes, and peroxisomes (Fig. 3c), and the third pellet (Fig. 3d) was a typical microsomal fraction, constituted by endoplasmic reticulum and plasma membrane fragments.

## Discussion

When coupled to ^99m^Tc, cells can be labeled with a relatively inexpensive and widely available radionuclide. Many studies show the feasibility of labeling stem cells with ^99m^Tc to track their homing [10, 15–21]. Usually, molecules such as peptides and antibodies are labeled with ^99m^Tc in order to become probes to monitor cell migration.

There are many mechanisms of labeling molecules. Essentially six major methods are employed in the preparation of labeled compounds for clinical use. The majority of cell labeling methods use a chelating agent. Here, we used a direct method of labeling with high cell viability and stability rates [15, 17–20, 22–25].

Radionuclide cell labeling has already been applied for tracking cells in cell therapy for myocardial infarction [30–32], cirrhosis [20], and acute stroke [18, 19, 33] in humans. The 6-h half-life of ^99m^Tc is an important advantage over the half-life of 110 min of [18F]FDG. 111-Indium-oxine, another commonly used radiopharmaceutical, allows cell tracking for up to 96 h but has disadvantages that include suboptimal photon energies, low-resolution images, and the requirement of an 18- to 24-h interval between injection and imaging [34, 35]. ^99m^Tc allows imaging for 24–48 h and results in higher image resolution and a lower radiation burden to the patient [34, 35].

Previous work by our group evaluated the binding sites of ^99m^Tc to mononuclear leukocytes since radiolabeled leukocytes have a potential for clinical use in detecting sites of inflammation [24]. In this work, we analyzed the binding sites of ^99m^Tc to BMMCs, which have a different composition when compared with peripheral blood. ^99m^Tc-labeled bone marrow mononuclear fraction has been widely used in cell therapy experiments [17–20] with the objective of tracking them after systemic infusion. Moreover, our findings were improved by the addition of electron microscopy data.

One of the most famous and most used radiopharmaceuticals to label leukocytes is ^99m^Tc- hexamethylpropyleamine oxime (HMPAO). Some studies showed that ^99m^Tc-HMPAO has high selectivity for eosinophils from blood compared with neutrophils and other blood leucocytes [26, 36]. In these cases, it was also shown that ^99m^Tc-HMPAO is stored in the eosinophils mainly in nuclear and cytoplasmic compartments.

As a crucial step to understanding the mechanism of radiopharmaceutical localization in a specific target organ for cell therapy, the interaction of the ^99m^Tc with bone marrow cells was evaluated. Our results showed that this is an intracellular labeling procedure that has cytoplasm, ribosomes unbound to membrane, and soluble molecules as targets. However, the natural interaction between ^99m^Tc to the cytosolic substrate still has to be elucidated.

Other radiopharmaceuticals, such as ^99m^Tc-methoxyisobutylisonitrile (MIBI) and ^99m^Tc-dimercaptosuccinic acid (DMSA), have had their binding sites studied before [27, 28, 37]. Similar to our results, data from whole heart preparations, largely derived from differential centrifugation techniques, indicated that most of ^99m^Tc-MIBI appears to be associated with the cytosolic fraction. In this study, especially designed to compare results of subcellular fractionation obtained with ^99m^Tc-(N-ethoxy,N-ethyl dithiocarbamato) nitrido (N-NOEt) and ^99m^Tc-MIBI, interesting results emerged [37, 38]. The fact that ^99m^Tc-N-NOEt activity was not released into the cytosol after membrane and organelle disruption suggested that this lipophilic marker remained tightly bound to the hydrophobic components of the cells. On the other hand, the monocationic ^99m^Tc-MIBI complex, in the same situation, was removed from disrupted mitochondria and transferred to the aqueous cytosolic phase. However, experiments that use subcellular fractionation procedures should be observed with caution because of dependence of the results on two important parameters: time of homogenization and centrifugation rates. In the mentioned study, if centrifugation time was extended to 180 sec, it was observed that approximately 70 % of ^99m^Tc-MIBI activity was released to cytosolic fraction as a result of disruption of mitochondria [38]. This result is in agreement with those reported by Crane et al. [37].

Subcellular distribution of ^99m^Tc-DMSA (dimercaptosuccinic acid) complex in the rat kidney has also been studied. One hour after intravenous injection of labeled DMSA, kidney tissue homogenate preparations were subjected to differential subfractionation to obtain cell organelles. Radioactivity distribution in relation to total radioactivity of kidney homogenate obtained in five repeated experiments was similar to our results [27, 28].

## Conclusions

In conclusion, the mechanisms involved in the cellular uptake of ^99m^Tc in bone marrow cells are not known, but the fact that the activity is distinctly localized in unique compartments indicates some specificity and not just a general distribution among structures.

Molecular imaging is undergoing constant change and is rapidly expanding. It spans all current life sciences and is being used at the frontiers of modern research. For the clinical radiologist, the future will bring applications of molecular imaging techniques into the standard diagnostic workflow.

## Abbreviations

BMMC: Bone marrow mononuclear cell
DAPI: 4'6-Diamidino-2-phenylndole
DMEM: Dulbecco’s modified Eagle’s medium
DMSA: Dimercaptosuccinic acid
[18F]FDG: 18F-Fluorodeoxyglycose
HMPAO: Hexamethylpropyleamine oxime
MIBI: Methoxyisobutylisonitrile
N-NOEt: (N-ethoxy,N-ethyl dithiocarbamato) nitrido
PBS: Phosphate-buffered saline
^99m^Tc: 99m Technetium
